# Progressive Insights into Metal-Organic Frameworks and Metal-Organic Framework-Membrane Composite Systems for Wastewater Management

**DOI:** 10.3390/molecules29071615

**Published:** 2024-04-03

**Authors:** Jilong Han, Hanya Zhang, Yuheng Fan, Lilong Zhou, Zhikun Zhang, Pengfei Li, Zhengjie Li, Yongsheng Du, Qingfen Meng

**Affiliations:** 1 College of Chemical and Pharmaceutical Engineering, Hebei University of Science and Technology, Shijiazhuang 050018, China; hanjilong@hebust.edu.cn (J.H.); zhy264835@163.com (H.Z.); fyh000401@163.com (Y.F.); lanruohe@126.com (L.Z.); zqz5437@psu.edu (Z.Z.); pengfeili@hebust.edu.cn (P.L.); 2 Qinghai Provincial Key Laboratory of Geology and Environment of Salt Lakes, Qinghai Institute of Salt Lakes, Chinese Academy of Sciences, Xining 810008, China; 3 Qinghai Qaeidam Xinghua Lithium Salt Co., Ltd., Golmud 817000, China; qqm23@sohu.com

**Keywords:** water resources, wastewater, metal-organic frameworks (MOFs), MOF-membrane composite

## Abstract

The sustainable management of wastewater through recycling and utilization stands as a pressing concern in the trajectory of societal advancement. Prioritizing the elimination of diverse organic contaminants is paramount in wastewater treatment, garnering significant attention from researchers worldwide. Emerging metal-organic framework materials (MOFs), bridging organic and inorganic attributes, have surfaced as novel adsorbents, showcasing pivotal potential in wastewater remediation. Nevertheless, challenges like limited water stability, elevated dissolution rates, and inadequate hydrophobicity persist in the context of wastewater treatment. To enhance the performance of MOFs, they can be modified through chemical or physical methods, and combined with membrane materials as additives to create membrane composite materials. These membrane composites, derived from MOFs, exhibit remarkable characteristics including enhanced porosity, adjustable pore dimensions, superior permeability, optimal conductivity, and robust water stability. Their ability to effectively sequester organic compounds has spurred significant research in this field. This paper introduces methods for enhancing the performance of MOFs and explores their potential applications in water treatment. It delves into the detailed design, synthesis strategies, and fabrication of composite membranes using MOFs. Furthermore, it focuses on the application prospects, challenges, and opportunities associated with MOF composite membranes in water treatment.

## 1. Introduction

With the swift progression of science and technology and the burgeoning global population, the demand for water has surged dramatically. While Earth boasts vast water reserves, a mere 2.5% of this constitutes fresh water accessible for direct human consumption. Consequently, the looming water scarcity has catapulted to the forefront of global concerns. Compounding this crisis is the unchecked discharge of wastewater from industrial and agricultural activities, which intensifies global water resource depletion [1,2,3]. This unchecked release has precipitated acute water shortages in numerous regions [4,5].

**Figure 1:**
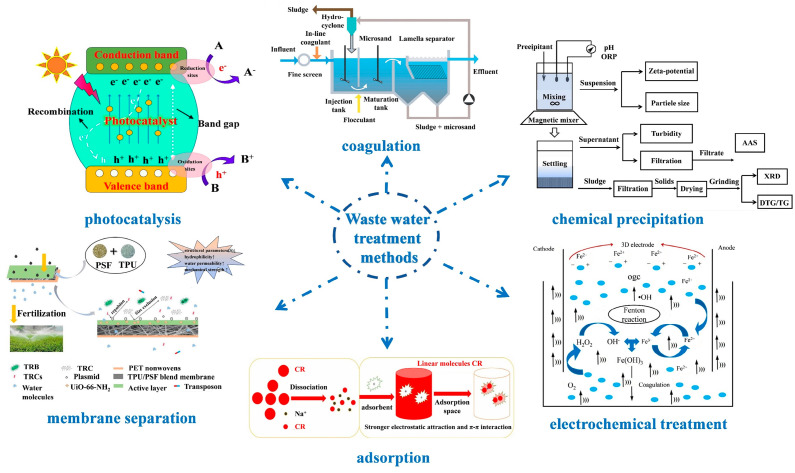
Schematic of Waste water treatment methods: photocatalysis [13], Copyright 2021, Elsevier. Coagulation [16], Copyright 2020, Royal Society of Chemistry. Chemical precipitation [19], Copyright 2018, Elsevier. Electrochemical treatment [25], Copyright 2020, Elsevier. Adsorption [28], Copyright 2021, Elsevier. Membrane separation [29], Copyright 2020, Elsevier. Reprinted/adapted with permission from Refs. [13,16,19,25,28,29].

Addressing this water scarcity necessitates innovative solutions, with the harnessing and repurposing of wastewater emerging as a pragmatic approach. Viewed through this lens, wastewater transforms into a prospective reservoir of “new” potable water. When judiciously treated, it can cater to diverse consumption needs, offering tangible social, environmental, and economic dividends [6]. To date, researchers have pioneered an array of wastewater treatment methodologies, as delineated in Figure 1. These encompass techniques like photocatalysis [7,8,9,10,11,12,13], coagulation [14,15,16,17,18], chemical precipitation [19,20,21,22,23], electrochemical treatments [24,25,26,27], adsorption [28], and membrane separation [29].

Among various wastewater treatment methodologies, adsorption stands out and has been extensively employed due to its superior efficiency, straightforward management, and user-friendly operation. The quintessential adsorbents typically exhibit these characteristics:

(i) Non-reactivity with both adsorbates and the medium;
(ii) Robust chemical, thermal stability, and mechanical resilience;
(iii) Expansive specific surface area combined with an apt pore and functional surface structure;
(iv) Pronounced affinity towards adsorbates;
(v) Ease of preparation and regeneration.

To date, a plethora of materials, including chitosan, activated carbon, zeolites, graphene, carbon nanotubes, hydrogel, and metal-organic frameworks (MOFs), have been investigated for wastewater treatment applications [30,31,32,33,34,35,36,37,38,39,40]. Activated carbon and zeolites are the most widely used adsorbents for wastewater treatment in industrial scale and carbon-based materials have also been deeply evaluated for their adsorption performance in organic pollutants removing; however, the limited adsorption capacity, difficult modification and regeneration, and high cost are the urgent problems restricting their large-scale application. Among these, MOFs, ingeniously crafted from inorganic metal centers and bridged organic ligands, are emerging as the frontrunners. The first MOF with permanent porosity was reported by Yaghi in 1995 [41], then Yaghi’s group synthesized MOF-5 (also known as IRMOF-1) with three-dimensional framework who can still remain its porous structure after removing guest molecules in 1999 [42]. Owing to the unique structural features of ultrahigh porosity and specific surface area, researchers have paid tons of attention to develop new kinds of MOFs and expand their application area in the last two decades. Remarkably, over 20,000 variants of MOFs have been synthesized using diverse techniques, which can be divided into several series of MOFs including IRMOF-n (isoreticular metal-organic framework), ZIF-n (Zeolitic Imidazolate Frameworks), MIL-n (Materiel Institut Lavoisier), UiO-n (University of Oslo), CAU-n (Christian Albrechts University), PCN-n (Porous Coordination Network), UTSA-n (University of Texas at San Antonio), NOTT-n (University of Nottingham), BUT (Beijing University of Technology), BUC-n (Beijing University of Civil Engineering and Architecture), ZJU-n (Zhejiang University), FJI-n (Fujian Institute of Research on Structure of Matter), and so on [43,44,45,46,47]. Their appeal lies in their distinctive structural attributes: vast specific surface area, generous pore volume, enhanced functionality, design flexibility, and a plethora of adsorptive sites [48,49,50,51,52,53]. Moreover, this vast MOFs library offers a treasure trove of potential ideal adsorbents tailored for specific contaminants. Especially, MOFs with excellent water stability (i.e., MIL-68, MIL-101, ZIF-8, UiO-66, and UiO-67) has further propelled their use in purging various pollutants from water systems [54,55,56,57,58,59,60,61,62,63,64].

**Figure 2:**
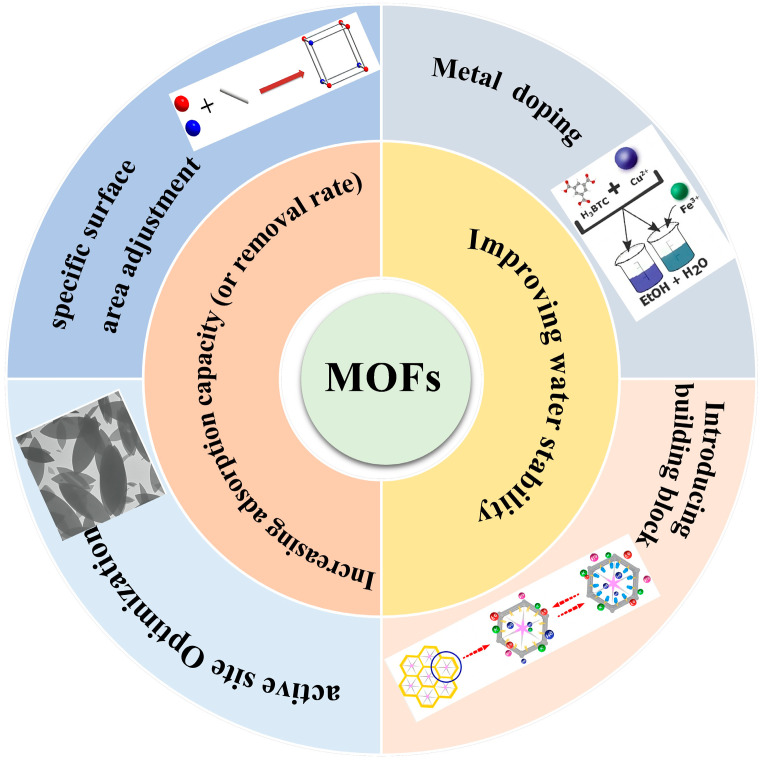

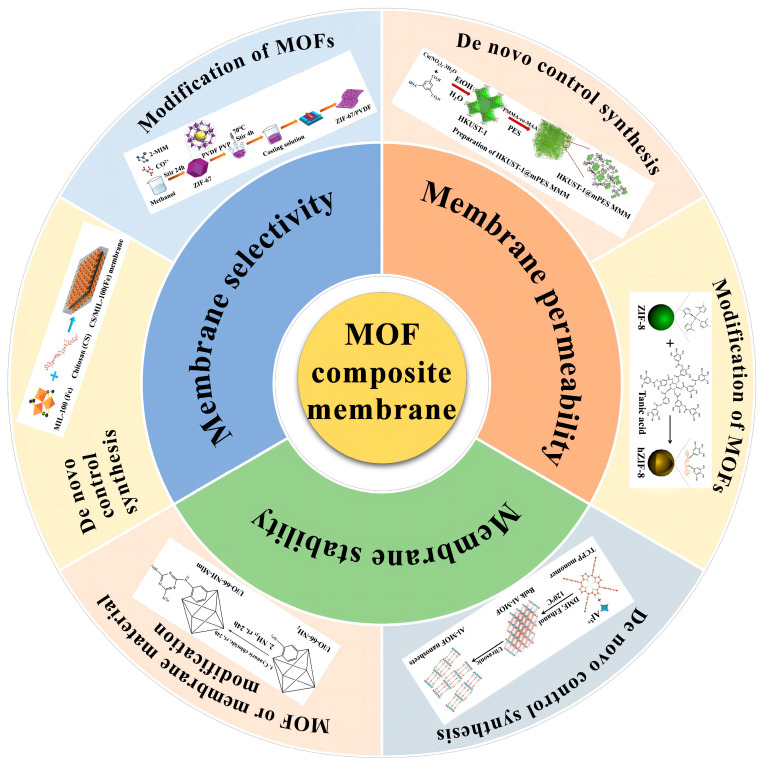
Feasible strategies for improving the stability and adsorption performance of MOFs and MOF composite membrane.

However, despite MOFs’ unparalleled adsorption prowess, challenges persist. Complex synthesis routes, suboptimal yields, and elevated raw material costs impede their widespread adoption in water treatment [65]. Numerous strategies are currently under exploration to amplify their efficacy and aqueous stability, as depicted in Figure 2.

Shifting focus to membrane separation, it has carved a niche in industrial wastewater treatment [66,67]. Commonly used membranes include microfiltration, ultrafiltration, nanofiltration, and reverse osmosis. By marrying the porosity and diversity of MOFs with the permeability and selectivity of membranes, MOF membrane materials are expected to become a functional membrane material with promising application prospects. These materials, characterized by no phase change, adaptability, and energy efficiency, are revolutionizing the separation of organic compounds in wastewater, marking a burgeoning research frontier.

This manuscript delves into the operational tactics, recent advancements of MOFs, and MOF-infused membranes in wastewater treatment, shedding light on the intricate adsorption mechanisms. It’s pivotal to underscore that augmenting the specific surface area and active sites can bolster MOFs’ adsorption capacity. Furthermore, introducing metals or organic entities can enhance stability. The strategic integration of MOFs with membranes and the subsequent refinement of MOF composite membranes can amplify their permeability, selectivity, and robustness. To put it briefly, this review aims to clarify methods to enhance the adsorption performance of MOFs and MOF composite membranes, and introduce examples for these methods in wastewater treatment. Besides, the topic of large-scale production of MOFs is also covered. At last, the application prospects, challenges, and opportunities of MOF and MOF-membrane composite in water treatment are thoroughly addressed.

## 2. Strategy for Boosting Adsorption Performance and Water Stability of MOFs

Building upon our previous discussions, the unique structural and surface attributes of MOFs have paved the way for their widespread application in wastewater treatment. Notably, the presence of diverse central metal sites and coordinately unsaturated metal sites introduces a novel method for the adsorption of toxic and hazardous substances. In recent years, the use of MOFs in wastewater treatment has garnered increased attention, thanks to the successful implementation of various design strategies. Through straightforward modifications or functionalizations, the performance of MOFs can be significantly enhanced. Concurrently, water stability remains a crucial factor influencing their industrial application, but it can be optimized using appropriate methods. This section highlights key factors that significantly influence the adsorption capacity (or removal rate) and water stability of MOFs.

### 2.1. Enhancing Adsorption Capacity or Removal Rate

The adsorption capacity (or removal rate) stands as a pivotal parameter when evaluating the performance of adsorbents. It directly impacts the adsorbent dose, subsequently influencing the investment required for the adsorption process. Given the relatively higher cost associated with MOFs, there’s a pressing drive to boost their adsorption capacity for contaminants in aqueous solutions. To this end, researchers have devised various methods, including enhancing the specific surface area and increasing active sites.

#### 2.1.1. Adjusting the Specific Surface Area

The specific surface area of porous materials plays a crucial role in shaping their adsorption performance. Consequently, there has been extensive research aimed at modulating the specific surface area of MOFs to bolster their adsorption capacity. This can be achieved through techniques such as metal doping, enlarging pore structures, and etching.

(1) Metal doping

**Figure 3:**
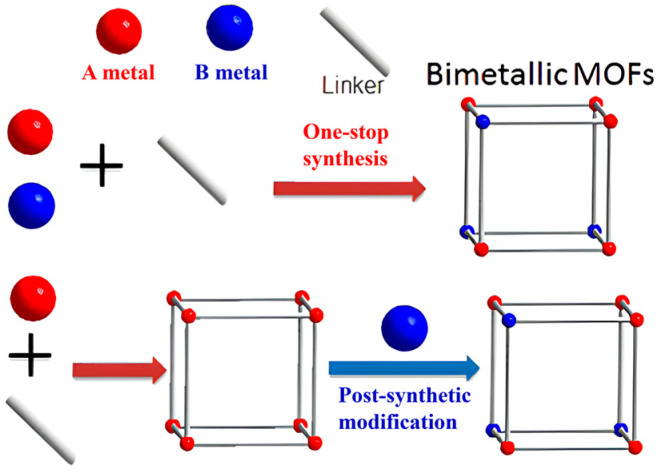
The preparation methods of bimetallic MOFs [69]. Copyright 2017, American Chemical Society. Reprinted/adapted with permission from Ref. [69].

To enhance the specific surface area of MOFs, an important strategy involves introducing new secondary metal nodes into the framework. This process typically entails incorporating metal sources (such as metal elements or metal nanoparticles) that can either reduce the crystallinity of the materials or modify their stacking arrangement or grafted on MOF, while maintaining the original topology. There are two methods for metal doping: (i) introduce metal ions into metal clusters to prepare bimetallic MOFs; (ii) prepare MOFs on the surface of metal nanoparticles or load metal nanoparticles into pores of MOFs. For the preparation of bimetallic MOFs, it can be categorized into two main types (illustrated in Figure 3), The first one is in-situ synthesis, in which both of the metal ions are simultaneously introduced into the mixture of solvent and organic linkers to prepare bimetallic MOFs through one-step method. The other method is the post-synthetic modification that the original metal ions of MOFs are partly replaced by a second metal ions through post-synthetic processes, also resulting in the formation of bimetallic MOFs. In bimetallic MOFs, there may be two metal clusters or one metal cluster with two metal ions, which can change the structural features and physicochemical properties of MOFs. What’s more, the ratio of the two metals can be adjusted to control the physicochemical properties of the bimetallic MOFs, offering flexibility in their design to match the requirements of specific application [68,69]. Generally, bimetallic MOFs show a higher specific surface area, together with that the introduced metal ions may change the surface charges or provide numerous active sites to enhance its adsorption performance for specific adsorbates.

**Figure 4:**
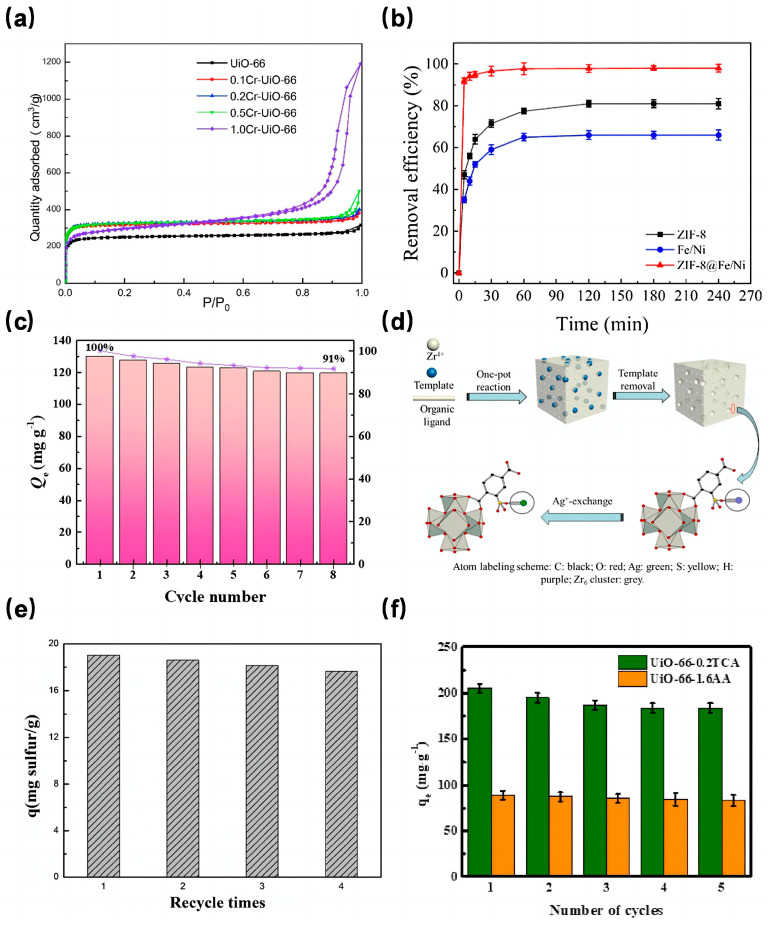
(**a**) Nitrogen adsorption (77 K) isotherms of UiO-66, 0.1Cr-UiO-66, 0.2Cr-UiO-66, 0.5Cr-UiO-66 and 1.0Cr-UiO-66 [70]; Copyright 2021, Elsevier. (**b**) Variation in removal efficiency of MG by ZIF-8, Fe/Ni and ZIF-8@Fe/Ni over time [73]; Copyright 2021, Elsevier. (**c**) The reusability of Fe_3_O_4_/ZIF-8 [75]; Copyright 2022, Elsevier. (**d**) Schematic diagram of HP-UiO-66-SO_3_H preparation and Ag^+^-exchange [80]; Copyright 2021, Elsevier. (**e**) Adsorption performance of HP-UiO-66-SO_3_Ag-0.375 for thiophene after regeneration [80]; Copyright 2021, Elsevier. (**f**) Regenerative adsorption performance test of UiO-66-0.2TCA and UiO-66-1.6AA [85]. Copyright 2020, Elsevier. Reprinted/adapted with permission from Refs. [70,73,75,80,85].

In a notable study, Fu et al. [70] synthesized Cr-doped UiO-66 with different Cr content by varying Cr content through the solvothermal method. The X-ray diffraction (XRD) peaks of these Cr-doped UiO-66 samples closely resembled those of pure UiO-66, suggesting that the metal doping process did not alter the crystal structure of UiO-66. As depicted in Figure 4a, the 0.2Cr-UiO-66 showcased the most expansive specific surface area, measuring 993 m^2^/g, marking a 31.6% increase compared to the unmodified UiO-66. Furthermore, water adsorption tests revealed that 0.2Cr-UiO-66’s water adsorption capacity significantly surpassed that of its pure counterpart. Similarly, Cao et al. [71] discovered that introducing cobalt ion (Co^2+^) enhanced the BET surface area and the total pore volume, although this enhancement diminished with increasing Co content. Dong et al. [72] observed that both the BET surface area and pore volume of Ce-UiO-67 exceeded those of UiO-67. This Ce-doped variant demonstrated a preference for cationic dyes, with adsorption capacities of 754.4 mg/g, 589.2 mg/g, and 191.6 mg/g for rhodamine B, malachite green, and methyl orange, respectively. This affinity can be attributed to the amplified electrostatic interactions resulting from Ce doping.

As discussed above, preparing MOFs on the surface of metal nanoparticles or loading metal nanoparticles into pores of MOFs are also effective ways to increase its surface area. ZIF-8, known for its excellent dispersion properties, has been a popular base material for crafting metal-doped adsorbents. For instance, Zhang et al. [73] integrated bimetallic Fe/Ni nanoparticles with ZIF-8 to produce the ZIF-8@Fe/Ni nanocomposite, which was then tested for its malachite green (MG) adsorption efficacy. This composite boasted a specific surface area of 919.050 m^2^/g, substantially larger than both ZIF-8 (246.370 m^2^/g) and Fe/Ni (32.350 m^2^/g). Adsorption tests (Figure 4b) indicated that ZIF-8@Fe/Ni achieved a remarkable 99% MG removal rate, considerably outperforming ZIF-8 (80%) and Fe/Ni nanoparticles (66%). This underscores the premise that enhancing the specific surface area can indeed elevate the removal rate. Notably, ZIF-8@Fe/Ni’s removal rate only dipped to 73% after five complete cycles, showcasing its sustained efficiency.

Addressing the challenges of limited recyclability and cumbersome solid-liquid separation associated with powder-state MOFs, Jiang et al. [74] crafted the Fe_3_O_4_@ZIF-8 core-shell magnetic composite material with an impressive specific surface area of 724.7 m^2^/g using the solvothermal method. This composite exhibited a high adsorption capacity for Pb^2+^ and Cu^2+^ in water and could be effortlessly separated from the solution using a magnetic field. Apart from preparing core-cell composite with magnetic nanoparticles, some researchers firstly prepared nanoparticles and MOFs separately, and then mixed them at a certain temperature and time to make the nanoparticles embeded into pores of MOFs. For example, Qu et al. [75] employed the magnetic material Fe_3_O_4_ to modify ZIF-8, resulting in the Fe_3_O_4_/ZIF-8 composite with a specific surface area of 1120.69 m^2^/g. This composite could adsorb phenol up to 129.8 mg/g (with a removal rate of 91%). As demonstrated in Figure 4c, even after eight adsorption-desorption cycles, the phenol removal rate remained at a commendable 91%. This suggests that magnetic MOFs adsorbents, besides amplifying the specific surface area, also deliver outstanding adsorption capacities and exhibit commendable reusability.

(2) Hierarchical MOFs

In order to remove pollutants with huge molecule size from wastewater, enlarging the layered size and pore size of MOFs have been regarded as one of the effective strategies to enhance the adsorption capacity. The pores of MOFs can be expanded to mesoporous or even macroporous pores by several strategies, which is called hierarchical MOFs. The synthetic strategies for hierarchical MOFs include template method, ion exchange method (linker labilization), sequential hydrothermal processes, and so on. The unique pore structure endow hierarchical MOFs a promising adsorbent with good performance. Their mesoporous and marcporous pores facilitate the diffusion of large molecules into MOF’s cages and provide enough space to restore them, while micropores in hierarchical MOFs control the size selectivity of guests accessing the immobilized molecules in mesopores [76,77]. For instance, Yuan et al. [78] introduced an innovative technique, termed “linker labeling”, designed to augment both the porosity and pore size of MOFs, thereby facilitating a layered pore structure. Building on this, Cheng et al. [79] employed a template method to craft a hydrophobic layered metal-organic framework (HZIF-8) that encompasses micropores, mesopores, and macropores.

Fast forward to 2021, Zhang et al. [80] pioneered the synthesis of a stratified pore UiO-66. In their approach, HP-UiO-66-SO_3_H was first produced using a template self-assembly method. Subsequently, HP-UiO-66-SO_3_Ag was derived through an ion exchange technique (as illustrated in Figure 4d). Impressively, the specific surface area of HP-UiO-66 SO_3_Ag-0.125 reached 1535 m^2^/g, marking an 82% enhancement compared to the unmodified UiO-66. This clearly indicates the efficacy of their method in elevating the specific surface area. Further experimental evaluations revealed that this modified MOF exhibited a robust adsorption capacity for compounds like thiophene and benzothiophene, coupled with commendable reusability (as depicted in Figure 4e). Besides, porous materials derived from hierarchical MOFs also attracted many attention for their application in wastewater treatment. Poudel et al. [81] prepared a Co-Al layered double hydroxide 3D porous carbon nanofiber (Co-Al-LDH@Fe_2_O_3_/3DPCNF) via the hydrothermal method, in which the Co-Al-LDH was derived from Co-MOFs. Co-Al-LDH@Fe_2_O_3_/3DPCNF has high porosity and a high specific surface area. The adsorption test results demonstrated that its maximum adsorption capacities for Cr(VI) and Pb(II) in water are 400.40 mg/g and 426.76 mg/g, respectively, with a very short adsorption saturation time.

In conclusion, the synthesis of multi-layered porous MOFs offers a promising avenue to bolster adsorption capacity. However, a general trend observed is that a smaller average pore diameter often corresponds to a larger total specific surface area. This relationship poses challenges in precisely controlling the pore size within these multi-layered structures.

(3) Etching

Etching is a simple and useful method to increase the specific area of MOFs without affecting their intrinsic crystal structure. The etching process can be achieved by the removal of junctions or clusters of metal ions through chemical processes, rendering MOFs more defective and endowing them with additional beneficial properties [82]. Delving into its applications, Hu et al. [83] innovatively employed phenolic acids both as surface functionalizing agents and etchants, leading to the synthesis of hollow MOFs. In a subsequent study, Xu et al. [84] crafted a defective amino-modified metal-organic framework, termed UiO-66-D-NH_2_, specifically designed to adsorb organic arsenic from aqueous mediums. Notably, the adsorptive affinity of UiO-66-D-NH_2_ for organic arsenic surpassed that of the original UiO-66 by a factor of 3.8. Moreover, when the initial concentration of organoarsenic was reduced to 1 mg/L, the removal rate exceeded 99.1%. This enhanced performance can be attributed to the synergistic effects of the defects and the -NH_2_ groups present in UiO-66-D-NH_2_.

Fast forward to 2021, a study focused on etching UiO-66 using a trio of monocarboxylic acids (MAS)-namely acetic acid (AA), trifluoroacetic acid (TFA), and trichloroacetic acid (TCA). This research aimed to discern the impact of crystal defect sites on the adsorption dynamics of dimethyl phthalate (DMP) and phthalic acid (PA) [85]. Among the various UiO-66 specimens, the one etched with AA at a concentration of 1.6 mol/L (dubbed UiO-66-1.6AA) boasted the most impressive specific surface area. This enhancement translated to a marked improvement in its adsorption capacity for both DMP and PA, registering increases of 18.05% and 41.59% respectively, compared to unetched UiO-66. Furthermore, regeneration studies underscored the material’s durability, with the adsorption capacities for PA and DMP on UiO-66-1.6AA remaining above 90% even after five cycles (as depicted in Figure 4f). Thus, introducing crystal defect sites via the etching method emerges as a potent strategy to bolster the adsorption efficacy of MOFs, especially concerning wastewater pollutants.

#### 2.1.2. Active Site Optimization

The density of active sites in porous materials plays a pivotal role in influencing their adsorptive properties. Recognizing this, researchers have dedicated extensive efforts to augment the number of active sites, aiming to enhance the adsorption capacity and removal rate of MOFs. A myriad of strategies have been employed in this pursuit, encompassing the construction of composites, the integration of functional groups, and the tailored shaping of MOFs to maximize their adsorptive active sites.

(1) Constructing composites

MOF-based composites, synthesized by integrating MOFs with other materials, have demonstrated enhanced performance compared to standalone MOFs and matrices. This enhancement is primarily due to the augmented adsorption sites provided by the composites.

**Figure 5:**
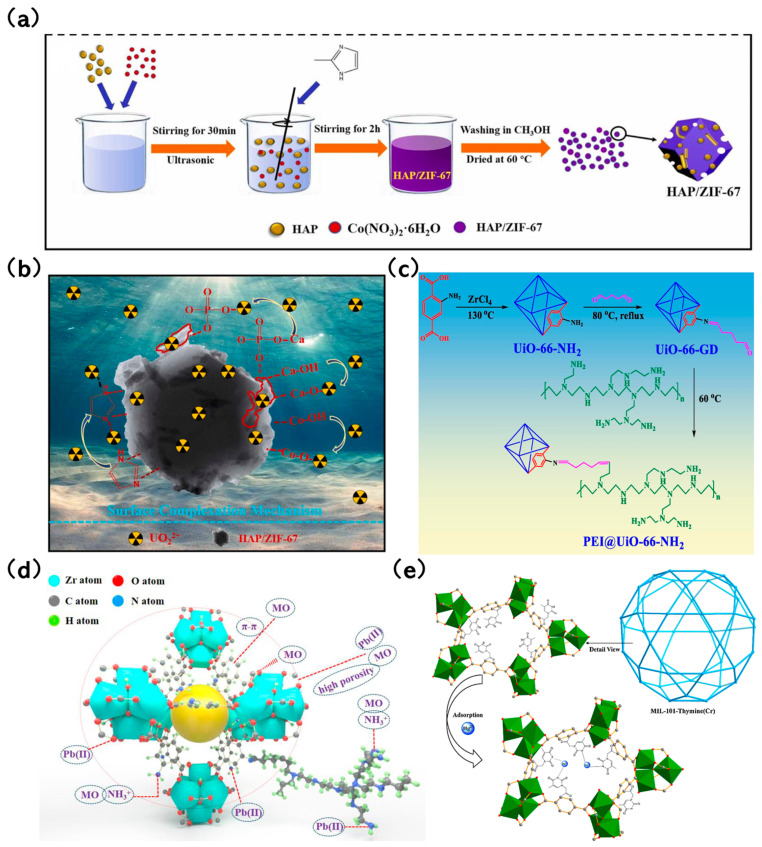
(**a**) Preparation method of HAP/ZIF-67 [87]; Copyright 2021, Elsevier. (**b**) The capture mechanism of U(VI) by HAP/ZIF-67 [87]; Copyright 2021, Elsevier. (**c**) Mechanism diagram of PEI grafting onto UiO-66-NH_2_ to obtain PEI@UiO-66-NH_2_ [92]; Copyright 2022, Elsevier. (**d**) Adsorption Mechanism of Pb(II) and MO on PEI@UiO-66-NH_2_ [92]; Copyright 2022, Elsevier. (**e**) Hg^2+^ is coordinated with the N of thymine on MIL-101-Thymine during Hg adsorption [95]. Copyright 2015, Elsevier. Reprinted/adapted with permission from Refs. [87,92,95].

Lv et al. [86] developed a layered nanoscale hydroxide-modified MOF-76 (LDH@MOF-76) composite. Its adsorption capacity for U(VI) reached 433.91 mg/g, marking a performance that’s twice as effective as pure LDH and 1.5 times that of MOF-76. This increased capacity is attributed to the additional hydroxy groups from the loaded LDH, which facilitate U(VI) adsorption. In a study by Xuan et al. [87], nanoscale hydroxyapatite (HAP) was utilized to craft a HAP/ZIF-67 composite using an ultrasound-assisted method (Figure 5a). This composite was designed for U(VI) removal from aqueous solutions. Impressively, the adsorption capacity of HAP/ZIF-67 for U(VI) was recorded at 453.1 mg/g, outperforming both HAP and ZIF-67 by 2.55 and 1.78 times, respectively. The high adsorption rate is largely due to the Ca-OH and PO_4_^3−^ groups in HAP (Figure 5b), which serve as binding sites for U(VI). Min et al. [88] highlighted that the U(VI) adsorption capacity of the nanoscale magnetic Fe_3_O_4_-modified ZIF-8 (Fe_3_O_4_@ZIF-8) was an impressive 539.7 mg/g at a pH level of 3. This efficiency is credited to the abundant functional groups present in Fe_3_O_4_@ZIF-8. Masoumi [89] encapsulated phosphotungstic acid (PTA) into MIL-53(Fe) to produce the PTA@MIL-53(Fe) composite using a one-pot method. The composite’s adsorption capacity for tetracycline hydrochloride was a staggering 1250 mg/g. The integration of PTA expanded the flexible pores of MIL-53(Fe), enhancing accessibility to the adsorption sites. Graphene oxide (GO), with its rich carboxyl groups and excellent dispersion capabilities, is deemed a fitting matrix for MOF combinations [90]. Rahimi and Mohaghegh et al. [91] crafted a Cu(tpa)/GO composite, where interactions between GO and Cu(tpa) were facilitated through π-π stacking and hydrogen bonding. This interaction allowed the residual groups of GO to serve as adsorption sites, forming coordination bonds with metal ions. As a result, the Cu(tpa)/GO composite showcased superior adsorption capacities for various metal ions compared to standalone Cu(tpa) and GO. To further amplify adsorption efficacy, dual modifications involving the introduction of functional groups and composite construction have been explored. For instance, Hu et al. [92] synthesized PEI@UiO-66-NH_2_ by grafting polyethyleneimine (PEI) onto UiO-66-NH_2_ (Figure 5c). The diverse active functional groups of PEI augmented the adsorption sites, leading to outstanding removal rates for Pb (II) and MO (Figure 5d).

In conclusion, the judicious selection of an appropriate matrix to harness combined advantages is pivotal in crafting high-performance MOF-based composites.

(2) Functionalization

Functionalization stands as a straightforward yet potent technique to enhance the surface properties of MOFs. Through this method, the physical or chemical attributes of MOFs can be tailored by introducing functional groups to organic ligands or by postsynthetic modifications tailored for specific applications. Moreover, these newly introduced functional groups can serve as novel adsorption sites or amplify the interaction with adsorbates, thereby boosting the adsorption efficacy. To date, over 20 distinct functional groups have been employed to craft functionalized MOFs, including hydroxy, carboxyl, nitro, amino, halogen atoms, sulfonic acid group, thiol groups, hydrosulphonyl, and organic substances containing these groups.

Li et al. [93] delved into the adsorption capabilities of MIL-101(Cr)-X (where X represents -Br, -CH_3_, -SO_3_H, -NO_2_, and -NH_2_) for tannic acid (TA). They discovered that -NO_2_ and -NH_2_ could form hydrogen bonds with TA molecules, altering the surface charges to amplify electrostatic interactions. Remarkably, the TA adsorption capacity of MIL-101(Cr)-NH_2_ soared to 2031 mg/g, outperforming a majority of other porous counterparts.

Ke et al. [94] crafted a series of thiol-functionalized CuBTC by leveraging the coordination bonding of coordinatively unsaturated metal centers in HKUST-1 with -SH groups present in dithioglycol. Adsorption experiments revealed that the integrated thiol groups served as potent adsorptive sites for Hg(II), boasting a high adsorption capacity of 714.29 mg/g. In contrast, the unmodified CuBTC showed no adsorption for Hg^2+^ under identical conditions.

Luo et al. [95] introduced an innovative thymine-functionalized MIL-101 (MIL-101-Thymine) tailored for mercury removal. The nitrogen in thymine within MIL-101-Thymine could coordinate with Hg^2+^, leading to its elevated adsorption capacity of 51.27 mg/g. In comparison, the adsorption capacity of MIL-101-NH_2_ stood at 30.67 mg/g. Additionally, MIL-101-Thymine showcased superior selectivity for Hg^2+^ over other cations, likely due to the highly selective interactions of T-Hg^2+^-T within MIL-101-Thymine (Figure 5e).

Subsequently, MIL-101(Cr) was enhanced through coordination bonding of unsaturated Cr metal centers with the -NH_2_ group in ethylenediamine (ED). The -NH_2_ groups within the pores of MIL-101(Cr) formed chelating binding sites, selectively adsorbing Pb(II). This modified version exhibited an adsorption capacity for Pb(II) ions that was fivefold that of standard MIL-101. In practical applications, ED-MIL-101 achieved an impressive 97.22% removal efficiency for Pb(II) ions [96].

Zhao et al. [97] developed an amidinothiourea-modified UiO-66-NH_2_ tailored for recovering Au(III) from aqueous solutions. Tests revealed its peak adsorption capacity for Au(III) was 227.68 mg/g, surpassing that of UiO-66-NH_2_, which was 166.23 mg/g. The underlying mechanism suggests that the sulfur and nitrogen groups in amidinothiourea serve as receptors for Au(III), forming stable complexes with Au(III) through chelation or complexation. In addition to the noble metal ions and heavy metal ions, a growing attention is now being paid to radioactive metal ions (including Sr, Cs, Co, Tc, U, Eu, I, etc.) from nuclear power plant and applications of radioisotopes (medicine, industrial activities, and agriculture) because of their high risks to environment and human [98]. For example, Sheha et al. performed a facile modification of strontium-based MOF using oxalic acid and the adsorption performance of the obtained MTSr-OX MOF for ^152+154^Eu radioisotopes was thoroughly evaluated [99]. Compared to the pristine strontium-based MOF, MTSr-OX MOF has a higher BET surface. Additionally, the introduced oxalate strengthen the interaction with ^152+154^Eu and provide new adsorption sites, which improve the adsorption capacity of ^152+154^Eu together. The maximum adsorption capacity of ^152+154^Eu on MTSr-OX MOF was 234.72 mg/g, almost four times higher than the pristine strontium-based MOF.

In summary, the current approach of refining MOFs-based porous materials by grafting diverse organic functional groups-either by modifying organic ligands or employing coordination chemistry-offers a straightforward and energy-efficient pathway. This paves the way for myriad opportunities in pollutant removal from aqueous solutions.

(3) Shape adjustment

**Figure 6:**
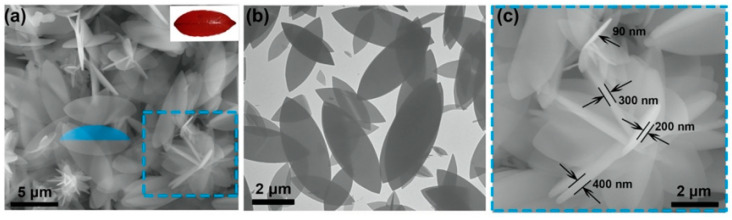
(**a**) SEM images of 2D ZIF-L [101]; Copyright 2021, Elsevier. (**b**) SEM images of 2D ZIF-L [101]; Copyright 2021, Elsevier. (**c**) TEM images of 2D ZIF-L [101]. Copyright 2021, Elsevier. Reprinted/adapted with permission from Ref. [101].

Generally, the polyhedral structure of MOFs limits the contact with other substances, that is, the exposure of active sites is not enough. Hence, shape adjustment is an efficient route to provide more adsorptive sites by altering the shape of MOFs without adducing new ingredients. Several synthesis methods have been applied to prepare MOFs with different shapes and their adsorption performances for pollutants in wastewater were explored. Yang et al. [100] prepared monodisperse ZIF-8 crystals with five different morphologies by controlling the concentration of Sixteen alkyl three methyl bromide (CTAB). Huang et al. [101] prepared two-dimensional (2D) leaf-like ZIF-L adsorbents by precipitation and investigated its phosphate removal performance. A two-dimensional lobed structure of ZIF-L can be clearly observed by Scanning Electronic Microscopy (SEM) and Transmission Electron Microscope (TEM), as shown in Figure 6. The adsorption capacity of 2D ZIF-L for phosphate was 75.18 mg/g, two times higher than that of ZIF-8. It can be explained as that the two-dimensional sheet structure of ZIF-L lead to more active sites exposed on the surface. Shape adjustment, a simple and direct method to improve the adsorption performance of MOFs, has received some attention, but the research on shape adjustment and control is still in its infancy and requires more study.

### 2.2. Improving Water Stability

The structural stability of MOFs in aqueous conditions is one of the most crucial factors to take into account when using MOFs for the remediation of aqueous contaminants. However, the weak coordination bonds between the metal and organic linkers are easily attacked by water molecules resulting in their poor water-stability of MOFs, which severely limits their application in water purification. It is crucial to develop techniques for improving their water stability while also boosting the adsorption performance. Up to now, several different methods including De novo control synthesis, ligand functionalization, metal doping, and constructing composite. The above strategies are very helpful to modify the water stability of MOFs to meet the industrial request.

Due to the fact that MOFs are formed by self-assembly of organic ligands and metal ions through coordination bonds, the strength of the coordination bond significantly affect the stability of MOFs. Thus, choosing suitable metal ions and organic linkers to form strong bonds through De novo-controlled synthesis is a feasible method. Carboxylate-based ligands (e.g., aliphatic carboxylic acid, aromatic carboxylic acids, and mixed carboxylic acid ligands) and nitrogen-based ligands (e.g., pyrazolates, imidazolates, tetrazolates, and triazolates) have been used to synthesize water stable MOFs based on the hard-soft-acid-base-theory (HSAB) [102]. That is, the stable MOFs with robust chemical bonds can be generated by the reaction between hard base-hard acid and soft base-soft acid. For example, hard acid metal ions with high valence (including Fe(III), Al(III), Cr(III), Zr(IV), and Hf(IV)) are chosen to coordinate with hard base carboxylate-based ligands to create stable MOFs. Soft-base nitrogen donor ligands form the other kind of stable MOFs with the soft-acid metal ions (e.g., Cd(II), Cu(II), Pd(II), Ni(II), Co(II), and Zn(II). To date, a limited number of water-stable MOFs have been reported, such as MIL-53, MIL-68, MIL-100(Fe, Cr), MIL-101(Cr, Fe, Al), ZIF-8, CAU-1, and Zr-MOFs [103]. UiO-66, one of the most famous Zr-MOFs, is well known as its excellent water stability due to its higher coordination number of its secondary building units (Zr_6_O_4_(OH)_4_). Of course, the other Zr-MOFs also show good water stability due to the strong Zr-O bond. Nevertheless, the numbers of water-stable MOFs are still finite, which facilitates the researchers continuously develop new MOFs with good stability based on the above theory. For example, In 2017, Lan et al. [104] synthesized two new Al(III) carboxyl MOFs (AlTCS-1 and AlTCS-2) by adding HF (hydrofluoric acid) aqueous solution or formic acid to the mixture for preparing MIL-53(Al). The stable-experiments showed that AlTCS-1 and AlTCS-2 are stable over a wide pH ranging from 1 to 11, and AlTCS-1 is even stable in aqua regia solution for at least 24 h. Chen et al. [105] used a carboxylic acid ligand of H_8_tdhb with high coordination linkage (8-position) and abundant hydrophobic substituents (6 methyl groups) to coordinate with Cu^2+^ and the obtained product is namely BUT-155. BUT-155 showed excellent water stability that can retain its structural integrity after treatment in water at room temperature for 10 days or in boiling water for 24 h. Panagiotou et al. [106] chose Zr(IV) as metal center to prepare two new 2-D Zr-MOFs (i.e., UCY-13 and UCY-14) based on Zr_6_ secondary building units and angular dicarboxylate ligands, in which the ligands are H_2_HFPBBA (4,4′-(hexafluoroisopropylidene)) bis (benzoic acid) and H_2_OBA (4,4′-oxybis (benzoic acid)). Stability studies indicated that UCY-13 and UCY-14 exhibit significant stability in various organic solvents and aqueous solutions of a wide pH range (0 to 13) as well as in aqueous solutions of various ions including SO_4_^2−^, HSO_3_^−^, ClO_4_^−^, NO_3_^−^ and HCO_3_^−^. What’s more, they exhibit exceptional UO_2_^2+^ sorption capacities of 984 mg/g for UCY-13 and 471 mg/g for UCY-14, which can be attributed to that the UO_2_^2+^ ions are proposed to ligate the accessible donor atoms of the Zr6 SBUs in UCY-13 and UCY-14 and possibly to interact with the dicarboxylic ligands forming dimeric (UO_2_^2+^)_2_ species inside the pores.

#### 2.2.1. Metal Doping

**Figure 7:**
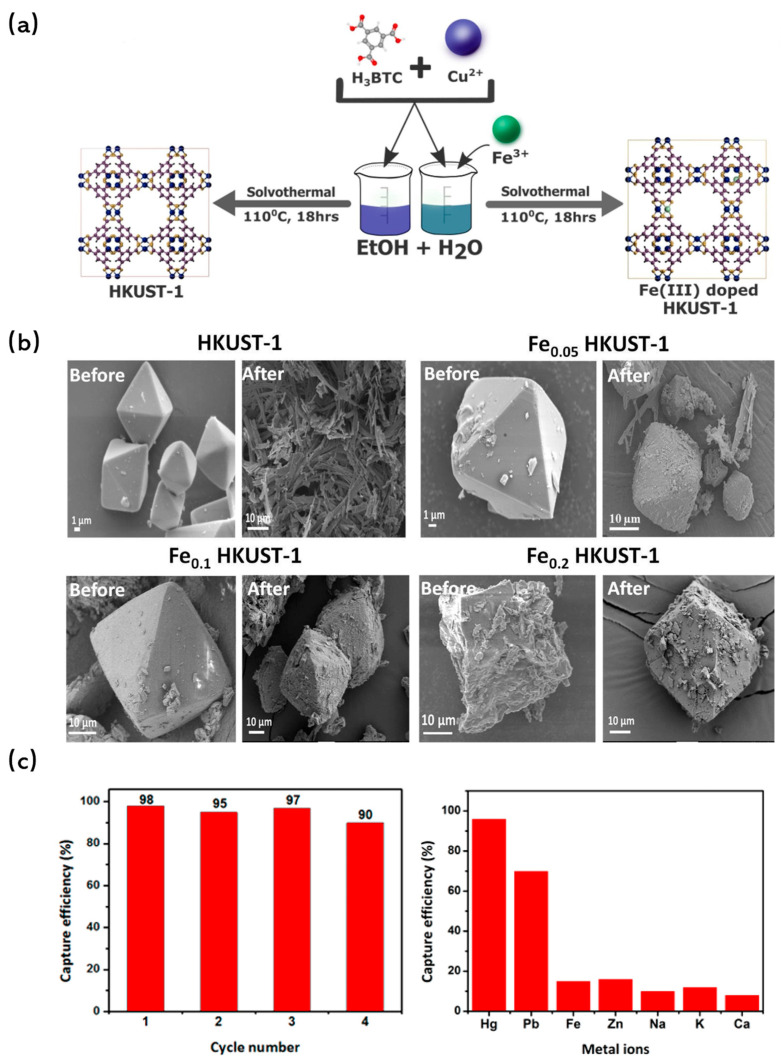
(**a**) Schematic for illustrating doping in HKUST-1 MOF [108]; Copyright 2021, Elsevier. (**b**) SEM images of HKUST-1 MOF, Fe_0.05_ HKUST-1 MOF, Fe_0.1_ HKUST-1 MOF and Fe_0.2_ HKUST-1 MOF before and after exposure to water for 2 h (MOF concentration of 1000 mg/L and pH 6.5) [108]; Copyright 2021, Elsevier. (**c**) Cycle performance for Hg(II) removal [109]. Copyright 2018, American Chemical Society. Reprinted/adapted with permission from Refs. [108,109].

Metal doping has been recognized not only as a method to increase the specific surface area of MOFs but also as a vital strategy to enhance their water stability. Li et al. [107] ventured into this domain by synthesizing Ni-doped MOF-5 through a solvothermal process. In this synthesis, Ni(II) ions were seamlessly incorporated into the framework, replacing a portion of the Zn(II) ions of [Zn_4_O]^6+^ clusters. Impressively, the resulting structure remained unaffected even after prolonged exposure to air, underscoring the enhanced stability of Ni-doped MOF-5. Building on this, Goyal et al. [108] synthesized Fe-doped HKUST-1 with varying doping concentrations using a one-pot solvothermal method (Figure 7a). Characterization techniques and simulations confirmed the successful incorporation of Fe into the framework by substituting Cu(II) sites. Notably, while the undoped HKUST-1 underwent morphological changes after a mere 2 h of water treatment, the Fe-substituted variant retained its structure even after 10 h, highlighting the stabilizing effect of the doped Fe ions (Figure 7b).

#### 2.2.2. Introducing Building Blocks

The spatial structure of MOFs, while intricate, is susceptible to dissolution and collapse in aqueous solutions. To address this vulnerability, researchers have turned to the introduction of rigid building blocks into the MOF pores, aiming to bolster their water stability. A case in point is the work by Jiang et al. [109] They initially synthesized the sulfur-based MOF, NENU-400, targeting mercury removal. However, the framework of NENU-400 was found to be unstable in mercury solutions. To overcome this, they introduced a molecular building block (MBBS) into the channels of NENU-400. The resulting modified NENU-400 not only exhibited enhanced water stability but also showcased a high adsorption capacity for mercury, further proving the efficacy of introducing building blocks (Figure 7c).

In order to facilitate the understanding and comparison of the properties of different MOF structures, all of the above literatures were classified and summarized, as shown in Table 1.

## 3. Application Strategy of MOF Membranes

To date, while MOFs exhibit promising water stability and high adsorption capacity, their industrial-scale applications remain limited. This is primarily due to their high cost and challenges associated with regeneration. In light of these challenges, membrane separation technology has emerged as a potential solution. For instance, membrane separation, as described by Pabby et al. [66], offers several advantages, including the absence of phase and chemical changes, excellent selectivity, adaptability, and low energy consumption. Given that the inherent porosity of MOFs can facilitate a sieving effect while ensuring high permeability, there has been a growing interest in the development of both bare MOF membranes and mixed matrix membranes (MMMs). In the latter, MOFs are incorporated into a polymer matrix. Unlike MOFs used solely for adsorption, MOF nanoparticles in MMMs serve a filtration function, eliminating the need for a desorption step. Moreover, their inclusion can bolster the water stability and specific surface area of the polymer membrane. With the evolution of innovative design strategies, the application of MOF-based membranes in wastewater treatment has gained significant prominence. This section delves into the primary factors influencing the permeability, selectivity, and stability of MOF-based membranes.

### 3.1. Bare MOF Membrane

Bare MOF membrane are prepared from pure MOF materials and therefore the properties of the MOF materials completely determine their permeability and selectivity [110].

#### 3.1.1. Growth of MOF Membrane on Unmodified Support

**Figure 8:**
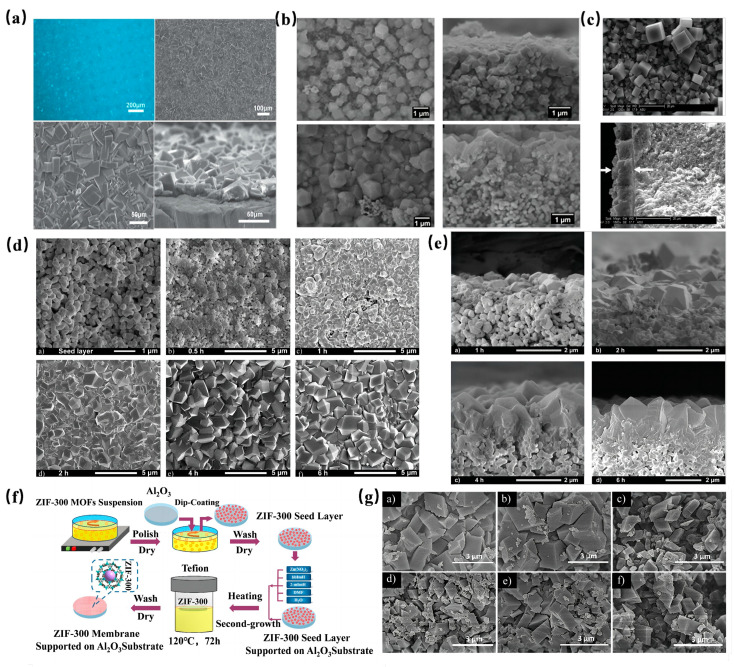
(**a**) Leica picture of the surface of the Ni_2_(L-asp)_2_ (bipy) membrane, SEM pictures of the surface of the Ni_2_(L-asp)_2_ (bipy) membrane and details of the densely packed crystallites and a cross-section SEM picture of the Ni_2_(L-asp)_2_ (bipy) membrane [111]; Copyright 2013, The Royal Society of Chemistry. (**b**) SEM images of ZIF-8 membrane grown for 2 min and for 30 min [112]; Copyright 2013, American Chemical Society. (**c**) SEM images of surface and cross-sectional morphology of MOF-5 thin membrane [113]; Copyright 2013, Elsevier. (**d**) a) SEM of ZIF-8 seed layer and membrane synthesized at 30 °C for b) 0.5 h, c) 1 h, d) 2 h, e) 4 h and f) 6 h [114]; Copyright 2011, Elsevier. (**e**) SEM pictures of the cross-section of ZIF-8 membrane synthesized at 30 °C for a) 1 h, b) 2 h, c) 4 h and d) 6 h [114]; Copyright 2011, Elsevier. (**f**) Schematic illustration of ZIF-300 membrane preparation process [115]; Copyright 2018, Elsevier. (**g**) SEM images of as- prepared ZIF-300 MOFs particles immersed in feed solutions at 25 °C for 30 days (a, CuSO_4_/H_2_O; b, CdSO_4_/H_2_O; c, CoSO_4_/H_2_O; d, RhB/H_2_O; e, MB/H_2_O; f, MO/H_2_O). The concentration of dye solution is 50 ppm, and the heavy metal ion solution are 10 mM [115]. Copyright 2018, Elsevier. Reprinted/adapted with permission from Refs. [111,112,113,114,115].

Before fabricating an MOF membrane, a suitable support is essential, as it offers a substrate for MOF crystal growth. Typically, hard metals serve as the membrane carriers. For instance, Kang et al. [111] employed a metallic nickel mesh as a support to synthesize a homochiral MOF membrane using the proto-growth method. SEM analyses revealed a robust connection between the MOF membrane, which had a thickness ranging from 20 to 30 mm, and the support (Figure 8a). Separation tests further demonstrated the membrane’s efficacy in separating a diol isomer mixture, achieving an ee value of 32.5%.

Among various metal supports, Al_2_O_3_ stands out as the most popular choice for bare MOF membranes. Its appeal stems from its attributes like high hardness, water insolubility, and temperature resistance. Kwon et al. [112] utilized custom-made α-Al_2_O_3_ disks as supports to fabricate an MOF membrane approximately 1.5µm thick using a reverse diffusion method (Figure 8b). In another study, Kasik et al. [113] adopted the secondary growth method with ball-milled MOF-5 seeds to produce an MOF-5 membrane on Al_2_O_3_ supports. SEM images (Figure 8c) showcased MOF-5 crystals of varying sizes and a membrane thickness of 10μm. However, during a p-xylene pervaporation test, the membrane’s p-xylene flux decreased to 70% of its initial value after 16 h, highlighting a potential fouling issue and suggesting a need for enhanced stability.

Addressing the stability concerns of MOF membranes on Al_2_O_3_ supports, some researchers have explored the synergy between more stable MOFs and Al_2_O_3_ to bolster membrane stability. Pan et al. [114] developed a novel ZIF-8 membrane on an alumina support using a hydrothermal seed growth (secondary growth) method. Observations (Figure 8d,e) indicated rapid growth upon exposure to the secondary growth solution. Impressively, after multiple cycles, the membrane’s permeability and separation coefficient variations were less than 2%, showcasing its commendable thermal stability. In a similar vein, Yuan et al. [115] synthesized a ZIF-300 membrane on an Al_2_O_3_ support for wastewater heavy metal ion removal (Figure 8f). Morphological examinations (Figure 8g) post a 30-day immersion in various solutions revealed minimal changes, attesting to the ZIF-300 membrane’s superior water stability. Furthermore, experimental outcomes highlighted an impressive water flux of 39.2 L/m^2^/h/bar and a CuSO_4_ rejection rate of 99.21%.

#### 3.1.2. Growth of MOF Membrane on Metal Supports

The growth and performance of MOF membranes can be significantly impacted by the weak bonding between MOF crystals and unmodified supports. To enhance this bond, it’s essential to modify the supports using appropriate techniques. For example, Weckhuysen et al. [116] utilized the layer-by-layer growth (LBL) method to fabricate a ZIF-8 thin membrane, employing gold-plated silicon wafers as carriers. Building on this, Zhu et al. [117] ventured into the synthesis of a ZIF membrane specifically designed for seawater desalination. They achieved this by modifying the surface of a ceramic carrier with biologically stimulated polydopamine (PDA). Both experimental and simulation data revealed that the resulting ZIF membrane possessed a small pore size, exhibited high stability in seawater, and demonstrated superior performance in seawater desalination. Notably, its ion rejection rate reached an impressive 99.8%, outperforming traditional zeolite membranes. In another innovative approach, Kasik et al. [118] crafted a ZIF-68 membrane, approximately 40 μm thick, using ZnO-modified α-alumina as a support. This was achieved through a modified reaction seed method. Pervaporation tests indicated that the xylene pervaporation flux of this membrane was roughly 5.4 times greater than that of the MOF-5 membrane.

### 3.2. MOF-Based Mixed Matrix Membrane

MOF-based mixed matrix membranes (MMMs) are crafted by integrating MOF nanoparticles into polymers or by coating the membrane surface with MOFs. This approach leverages the spatial structure and abundant pores of MOFs combined with the selective permeability of polymer membranes. Such a synergy not only addresses the trade-off between permeability and selectivity inherent in polymer membranes but also bolsters their mechanical strength and thermal stability [67]. Consequently, the fabrication and application of MOF-based MMMs in water treatment have emerged as pivotal areas in the industrial utilization of MOFs. This surge in interest has prompted the scientific community to prioritize research on MOF-based MMMs, with a focus on three primary challenges: membrane permeability, selectivity, and stability. This section delves into strategies to optimize the properties of MOF-based MMMs.

#### 3.2.1. Membrane Permeability

Permeability stands as a vital metric in assessing membrane performance. Higher permeability or flux translates to more robust net water movement. Numerous researchers have explored methodologies to enhance permeability, with strategies encompassing de novo control and MOF modifications.

##### De Novo Control Synthesis

**Figure 9:**
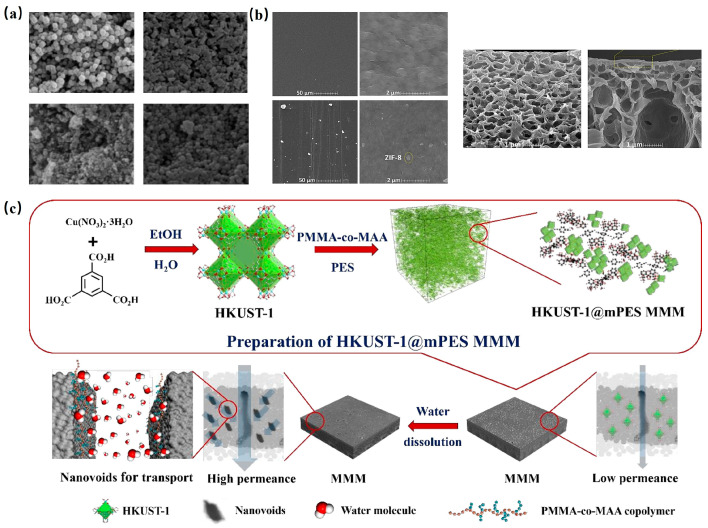
(**a**) SEM of ZIF-8, NH_2_-MIL-53(Al), MIL-53(Al), MIL-101(Cr) [119]; Copyright 2013, American Chemical Society. (**b**) Surface FE-SEM image of unmodified membrane and modified TFC membrane Cross-section FE-SEM image of unmodified membrane and modified TFC membrane [122]. Copyright 2018, Elsevier. (**c**) Schematic diagram of HKUST-1@mPES MMM prepared by nano-pore method and its application in water treatment [123]. Copyright 2019, American Chemical Society. Reprinted/adapted with permission from Refs. [119,122,123].

The structural attributes of MOFs and their compatibility with polymer membranes play a pivotal role in determining the permeability and selectivity of MMMs. As such, various MOFs have been investigated in tandem with different polymer membranes to augment permeability. For instance, Sorribas et al. [119]. crafted four MMMs through in situ interfacial polymerization, embedding MOF nanoparticles (like ZIF-8, MIL-53(Al), NH_2_-MIL-53(Al), and MIL-101(Cr)) into polyamide (PA) membrane layers, as depicted in Figure 9a. These MMMs exhibited enhanced permeability for MeOH and THF compared to pure PA membranes.

In a notable study, Li et al. [120] incorporated the hydrophilic MOF-801, known for its superior water absorption, into a chitosan (CS) matrix. This MOF-801/CS MMM was designed for ethanol percolation and dehydration, with the integrated MOF-801 significantly elevating the selective permeability to water. Pervaporation tests revealed a permeation barrier of 1200 g/m^2^/h and a high separation factor of 14.9.

Zirehpour et al. [121] developed MMMs by infusing nano-MOF particles, composed of silver (I) and 1,3,5-benzenetricarboxylic acid, into polyamide layers. Tests indicated that MMMs with a 0.04% MOF loading had a permeability 1.29 times that of a pure polyamide membrane.

Seeking to enhance desalination performance, Mohammad et al. [122] fabricated a thin-film composite (TFC) membrane by layering ultrathin ZIF-8 onto a polyvinylidene fluoride (PVDF) membrane. Air gap membrane distillation results showcased that the permeation barrier of the ZIF-8/PVDF membrane was a staggering 350% higher than its unmodified counterpart. SEM images (Figure 9b) attributed this high permeability to an increased pore count post-modification.

Lin et al. [123] introduced a novel polyethersulfone-based ultrafiltration membrane (HKUST-1@mPES MMM) by amalgamating HKUST-1 with poly (methyl methacrylate-co-methacrylic acid) (PMMA-co-MAA), as illustrated in Figure 9c. The resultant HKUST-1@mPES MMM exhibited a pure water permeability of up to 490 L/m^2^/h/bar, nearly tripling the performance of mPES without HKUST-1.

Lastly, Dai et al. [124] crafted an MOF composite membrane by integrating Cu-BDC nanosheets into a polyamide active layer via interfacial polymerization. The water permeation flux of this MMM saw an approximate 50% increase compared to the base membrane.

##### Modification of MOFs

The structure and composition of the incorporated MOFs play a pivotal role in determining the performance of MOF-based MMMs. Recognizing this, researchers have embarked on various MOF modification strategies, such as introducing new substances, functionalizing, refining synthesis techniques, and etching. These modifications aim to tailor MOF features, indirectly amplifying the permeability of MOF-based MMMs.

One effective strategy for MOF modification involves the introduction of new substances. A notable example is the work of Li et al. [125], who crafted MMMs by integrating superhydrophobic alkyl-modified MAF-6 (RHO-[Zn(eim)_2_]) into polydimethylsiloxane (PDMS) polymers. Pervaporation tests of ethanol/water mixtures revealed that these MMMs exhibited superior flux and separation factors compared to pure PDMS membranes, attributed to their hydrophilicity and high porosity. Another innovative approach was undertaken by Benzaqui et al. [126], who introduced polyethylene glycol (PEG) onto ZIF-8 nanoparticles to produce PEGylated ZIF-8 nanoparticles. These were then amalgamated with polyvinyl alcohol (PVA) to fabricate dense and supported MMMs. The resultant MMM showcased a permeation flux significantly higher than a pure PVA membrane, thanks to the molecular sieving effects of ZIF-8 and the membrane’s enhanced interfacial properties.

Functionalization and etching can alter the hydrophilic and hydrophobic properties of MOFs, potentially enhancing the compatibility of MMMs. Sun et al. [127] synthesized hydrophilic hollow ZIF-8 (hZIF-8) by surface functionalization using tannic acid (TA). This was then incorporated into a polysulfone (PSF) casting solution to produce a novel hybrid ultrafiltration (UF) membrane. The resulting membrane displayed a water permeability significantly higher than the base PSF membrane, attributed to the surface properties and nanostructure of hZIF-8. Liao et al. [128] employed a synergistic etching and surface functionalization mechanism using TA to etch ZIF-8, resulting in hydrophilic hollow nanocubes (HHNs) with abundant surface hydroxyl groups. These HHNs were then integrated into a polyamide (PA) layer of a nanofiltration membrane, producing a thin film nanocomposite (TFN) membrane with enhanced permeability and salt rejection. Capitalizing on the functional groups present in graphene oxide (GO) membranes, Ying et al. [129] developed a GO membrane intercalated with super-hydrophilic MOFs on a modified polyacrylonitrile (PAN) support. This was achieved using a novel pressure-assisted self-assembly (PASA) filtration technology. The resulting membrane exhibited impressive permeation flux and water content.

In conclusion, the permeability of MOF-based MMMs can be substantially enhanced by judiciously modifying MOFs and embedding them into membranes. Such advancements hold immense promise for wastewater treatment and various industrial separation processes.

#### 3.2.2. Membrane Selectivity

Selectivity stands as another pivotal criterion for evaluating membrane performance. Superior selectivity translates to heightened separation factors, adsorption capacities, and rejection rates. Crafting MMMs with MOFs has emerged as a promising avenue to enhance their selectivity. Various methods, including de novo control synthesis, MOF modification, and shape control, have been explored to elevate the selectivity of MMM membranes.

##### De Novo Control Synthesis

The characteristics of MOFs significantly influence the performance of MOF-based MMMs, impacting the selectivity of MMMs. Mao et al. [130] adeptly crafted MMMs with defect-free active layers through interfacial polymerization for ethanol pervaporation. This process led to a simultaneous increase in both the permeation flux and separation factor of the composite membrane. In another study, Ren et al. [131] integrated MAF-9 into a PDMS matrix to produce MAF-9/PDMS MMMs. The resulting composite membrane’s separation factor was 24% superior to that of the pure PDMS membrane. This enhancement was attributed to the superhydrophobic properties of the -CF_3_ groups in MAF-9, which predisposed the composite membrane to preferentially adsorb and permeate butanol. Abdelhameed et al. [132] embarked on a two-step process: first synthesizing a porous CA membrane, then crafting MMMs by in-situ synthesis of Cu-BTC within the CA membrane. Their findings revealed that the Cu-BTC/CA membrane’s adsorption capacity for dimethoate significantly outstripped that of the CA membrane.

**Figure 10:**
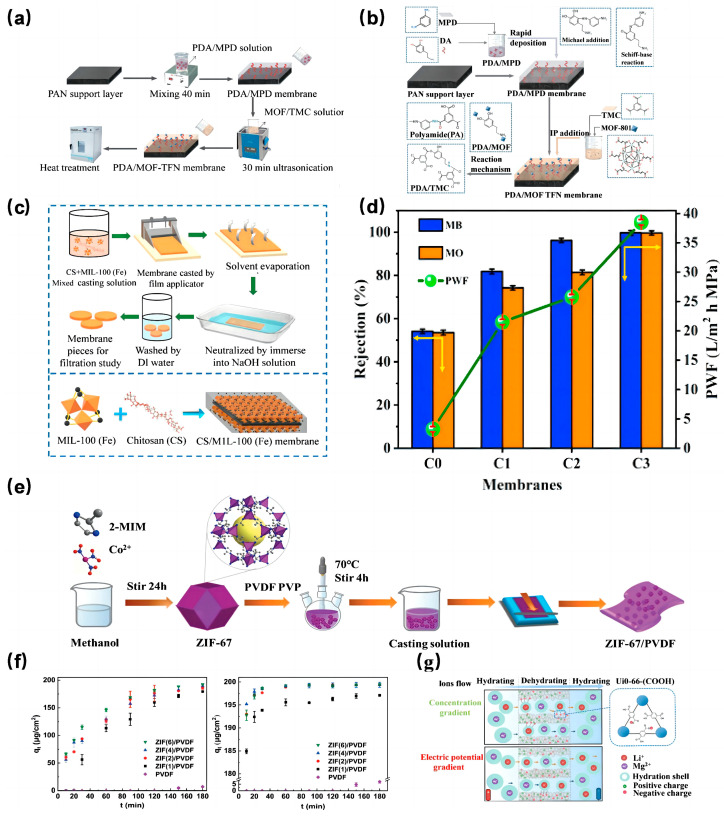
(**a**) Schematic of PDA/MOF-TFN membrane synthesis [134]; Copyright 2020, Elsevier. (**b**) Mechanism of the chemical reactions during interfacial polymerization [134]; Copyright 2020, Elsevier. (**c**) Schematic illustration of the fabrication of CS/MIL-100(Fe) membrane by the membrane casting technique [136]; Copyright 2021, Elsevier. (**d**) effect of MB and MO rejection, PWF at 0.4 MPa [136]; Copyright 2021, Elsevier. (**e**) Illustration of the formation process of the ZIF-67/PVDF hybrid membrane [135]; Copyright 2021, Royal Society of Chemistry. (**f**) Adsorption kinetics of MG and FA by pristine PVDF and the ZIF(x)/PVDF hybrid membranes [135]; Copyright 2021, Royal Society of Chemistry. (**g**) Schematic of a double-layered Zr-based membrane (UiO-66-(COOH)_2_/UiO-66-NH_2_ AAO membrane) for Li^+^ extraction from brine under different driving forces [138]. Copyright 2022, Elsevier. Reprinted/adapted with permission from Refs. [134,135,136,138].

Given the diverse contaminants present in industrial and domestic wastewater, researchers have probed the selectivity of MOF-based MMMs for various substances, including metal ions and dyes. He et al. [133] developed a thin film nanocomposite (TFN) membrane by incorporating UiO-66 nanoparticles of varying diameters into selective layers. Their results showcased impressive rejection rates for multiple anions using the TFN-0.15 wt% membrane. In a subsequent study, He et al. [134] introduced a novel polydopamine/metal-organic framework thin film nanocomposite (PDA/MOF-TFN) for forward osmosis (FO) (Figure 10a,b). This membrane exhibited a high rejection rate for several metal cations. Other researchers, such as Liu et al. [135] and Gnanasekaran et al. [136], have also made significant strides in this domain (Figure 10c–f), demonstrating the potential of MOF-based MMMs in wastewater treatment.

##### Modification of MOFs

The efficacy of MOF composite membranes largely hinges on the structure and composition of the incorporated nanomaterials. Recognizing this, researchers have sought to modify MOFs to indirectly enhance the performance of MOF-based MMMs. Golpour et al. [137] crafted a novel thin film nanocomposite (TFN) membrane by first synthesizing the water-stable, hydrophilic UiO-66-NH_2_ and then integrating it into a dense selective polyamide (PA) layer. Their findings underscored the enhanced thermal stability and mechanical strength of the composite membrane compared to the original. In another innovative approach, Xiao et al. [138] and Xu et al. [139] both successfully developed MOF membranes with high cation rejection rates (Figure 10g), showcasing their potential in extracting pure lithium and sodium ions from salt lakes and seawater.

##### Shape Control

Controlling the shape of MOFs can effectively amplify the number of active sites in MOF-based MMMs, thereby enhancing their selectivity. Ali et al. [140] pioneered the creation of three-dimensional (3D) membrane capsules, encapsulating UiO-66-(NH_2_)_2_ nanoparticles. Their results revealed impressive adsorption capacities for various metal cations. Similarly, Li et al. [141] developed a composite membrane featuring flower-like MIL-53-OH on a polyacrylonitrile/polyethyleneimine membrane. The unique structure and properties of the flower-like MIL-53-OH endowed the composite membrane with exceptional oil displacement hydration layers and a retention rate exceeding 99% for both dye and emulsified oil.

#### 3.2.3. Membrane Stability

For industrial applications, it’s imperative that membranes exhibit robust stability, ensuring sustained separation performance over extended periods. The fusion of MOFs with membranes has garnered significant attention as researchers seek to bolster membrane stability.

##### De Novo Control Synthesis

**Figure 11:**
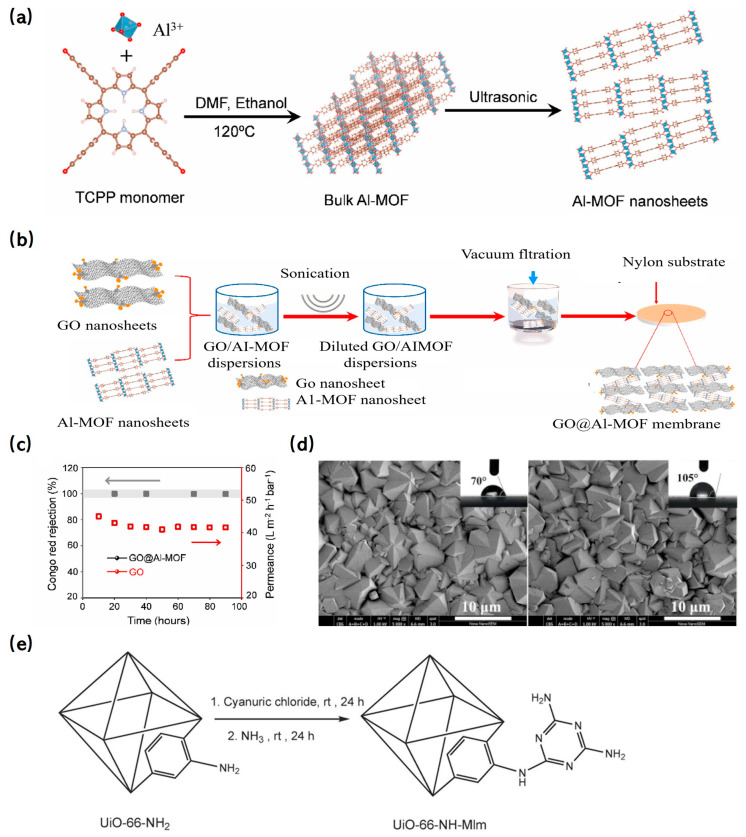
(**a**) Schematic illustration of the synthesis of Al-MOF bulk and nanosheets [142]; Copyright 2022, Elsevier. (**b**) Schematic illustration of the synthesis process of GO@Al-MOF composite membrane [142]; Copyright 2022, Elsevier. (**c**) Long-term separation stability of the GO@Al-MOF membrane and GO membrane in rejecting Congo red [142]; Copyright 2022, Elsevier. (**d**) Comparison of surface micrographs of pristine and modified ZIF-8 membrane, and corresponding static water contact angles [144]; Copyright 2017, Elsevier. (**e**) Preparation of UiO-66-NH-Mlm [145]. Copyright 2020, Elsevier. Reprinted/adapted with permission from Refs. [142,144,145].

MOF nanoparticles can be embedded within polymer membrane layers or adorned on the membrane’s surface, providing reinforcement that enhances stability. The diverse structural attributes of MOFs offer the potential to pair specific MOFs with particular membranes, optimizing both stability and separation performance. For instance, two-dimensional (2D) materials like GO membranes tend to swell in water and are characterized by non-uniform interlayer spacing, which compromises their stability and results in erratic permeability. To counteract these limitations, MOFs have been integrated with GO membranes. A notable example is the work of Alemayehu et al. [142], who crafted a composite membrane by incorporating porous 2D-Al-MOF nanosheets into a GO membrane. The synthesis schematic and preparation process are depicted in Figure 11a and 11b, respectively. Remarkably, the GO@Al-MOF composite membrane demonstrated a 99.9% rejection rate for Congo red and maintained this stellar performance consistently for 140 h, underscoring its superior stability (Figure 11c).

Thus, judiciously pairing MOFs with membranes can effectively mitigate the inherent limitations of either component. Furthermore, by modifying the MOF materials or membrane carriers, the properties of the composite membrane can be further enhanced.

##### MOF or Membrane Material Modification

The efficacy of MOF composite membranes is contingent not only on the structure and composition of the doped nanomaterials but also on the intrinsic attributes of the membrane. Consequently, modifying both MOFs and membranes emerges as a promising strategy to bolster the stability and overall performance of MOF composite membranes. For instance, Lu et al. [143] treated a PVDF membrane with a 25% ethylenediamine aqueous solution, introducing polar groups like -OH onto its surface. Subsequently, a highly stable MOF, NH_2_-MIL-53, was synthesized in situ on this modified PVDF ultrafiltration membrane. In another innovative approach, Zhang et al. [144] enhanced the hydrophobicity of a ZIF-8 membrane by substituting its methylimidazole ligand with the more hydrophobic 5,6-dimethylbenzimidazole. SEM and water contact angle analyses (Figure 11d) confirmed the improved hydrophobic properties post-modification. Golpour et al. [137] crafted a novel TFN membrane by incorporating synthesized UiO-66-NH_2_ into a dense selective polyamide (PA) layer atop a polyphenylsulfone (PPSU)-graphene oxide (GO) support layer. Their results highlighted the enhanced thermal stability and mechanical strength of the composite membrane. Similarly, Samari et al. [145] developed a polyethersulfone (PES) UF membrane with impressive thermal stability and permeability by integrating melamine-modified UiO-66-NH_2_ (Figure 11e), further enhancing the composite membrane’s stability.

In conclusion, the stability of MOF composite membranes can be significantly enhanced through strategic MOF modifications and their subsequent integration with membranes.

The applications of MOF compositions and MOF-membrane composite in water treatment is summarized, as shown in Table 2.

Nevertheless, the practical application of MOF composite membranes is not without challenges. For instance, polymer membranes often grapple with a trade-off between flux and separation factor. Furthermore, after prolonged operation, they can exhibit high swellability and diminished mechanical strength, which can precipitate a marked decline in their separation efficacy [146]. As such, there’s a pressing need for further exploration and refinement in the utilization of MOF composite membranes for wastewater treatment.

## 4. Discussion on Material Costs and Related Full-Scale Applications

As far as we know, the cost and output of MOFs are the primary factors limiting its large-scale industrial applications because that the common used solvothermal method is time-consuming with relatively low yield. Up to now, only a few MOFs are commercially produced by several international companies, typically at high prices [147]. Hence, there is a growing emphasis on developing green and cost-effective manufacturing processes to achieve sustainable production of MOFs. Researchers provide several kinds of strategies to overcome the above challenges: (i) utilizing inexpensive raw materials; (ii) minimizing solvents or employing environmental friendly alternatives; (iii) designing straightforward and efficient synthesis methods [147,148,149,150,151].

For instance, Bagi et al. [149] developed a low-cost and energy-efficient continuous manufacturing process for MOF-808-a Zr-MOF, in which a continuous flow reactor was used. Under optimal conditions, the N,N-dimethylformamide solvent and formic acid modulator volumetric amounts were decreased by 84% and 67%, respectively. This substantial reduction in material preparation costs resulted in a productivity of 95,155 kg m^−3^ day^−1^, which represents a two order of magnitude increase to that in batch (335.5 kg m^−3^ day^−1^). Moreover, the minimum cost of manufacturing MOF-808 in a laboratory-scale flow synthesis route was ~$3 per g, representing a significant decrease in cost compared to current MOF synthesis prices. Similarly, Boukayouht et al. [150] highlight another example meeting industrialization requirements, where the cost of 1 kg of MIL-53(Al) obtained from waste costs is only $3.49, which is significantly lower than that of the commercial alternatives (e.g., Basolite A100, priced at $10,000/kg). Furthermore, they emphasize the potential of utilizing natural and industrial waste by-products as sources of organic ligands and metal nodes for MOFs, underscoring the importance of developing green and sustainable synthesis methods conducive to large-scale production and industrial application. Severino et al. [151] highlight the potential of MIL-160 as a benchmark MOF for industrial series production and commercialization, and identify a simple method of calculating production costs by evaluating process parameters in MOFs costs (scale, cost of raw-materials, recirculation, washing etc.). The expected cost ranged from ca. 55 $/kg at 100 tons/year down to 29.5 $/kg for 1 kton/year production with longer term perspectives of reaching costs below 10 $/kg once the bio-derived ligand is considered for the large-scale production of bioplastics.

In conclusion, the industrial application of MOFs holds promise, and future research should not only pursue the advantages of advanced synthesis processes but also concentrate on developing methods better suited for sustainable pilot production and industrial applications of MOFs [147]. Although MOFs has good potential in industrial production, it is rarely used in actual production and life, and it is necessary to make it easier to recycle, low price and easy to regenerate through various methods to meet the needs of industrial applications.

## 5. Conclusions and Outlook

Addressing wastewater treatment remains a pressing concern, given the detrimental effects of untreated wastewater on human health and its indirect contribution to global water scarcity. Over the years, numerous methodologies have been explored to tackle this challenge. Among these, Metal-Organic Frameworks (MOFs) have garnered significant attention due to their adjustable pore structures, expansive specific surface areas, structural and functional diversity, and abundant unsaturated metal sites. Their high adsorption capacities make them particularly suitable for wastewater treatment. However, the challenge of regeneration, primarily due to recovery difficulties, persists.

To circumvent this, researchers have delved into integrating MOFs with membranes, resulting in MOF composite membranes. These membranes, characterized by their excellent compatibility and highly adjustable pore structures, have shown promise in wastewater treatment, especially given their high selectivity and permeability. Compared to conventional membranes, the enhanced permeability of MOF composite membranes contributes to improved desalination performance. The higher selectivity of MOF composite membranes aids in the removal of metal ions and dyes from water, as well as in oil-water separation. Additionally, the high water stability of MOF composite membranes also facilitates their application in water treatment. In summary, the improved performance of MOF composite membranes in various aspects underscores their significant potential for wastewater treatment. This review has provided insights into various types of MOF composite membranes and the strategies employed to enhance their stability, selectivity, and permeability.

Drawing from this comprehensive review, the future trajectory for MOF composite membranes can be summarized in the following key directions:

(i) Preparation and Modification: The synthesis of MOFs for composite membranes should prioritize enhancing their porosity, flexibility, and stability. When modifying these membranes, efforts should be directed towards achieving optimal membrane thickness and permeability, potentially aiming for even thinner membranes. Additionally, achieving a seamless bond between MOFs and the membrane is paramount.
(ii) Operational Stability: In real-world wastewater treatment scenarios, a membrane’s water stability is of utmost importance. Thus, when fabricating MOF composite membranes, emphasis should be placed on ensuring their long-term stability, especially in challenging aqueous environments such as those with strong acids, strong bases, or elevated temperatures.
(iii) Recyclability and Economic Viability: Post-wastewater treatment, the recyclability of MOF composite membranes emerges as a significant consideration. Beyond the environmental implications, economic factors play a pivotal role. As such, there’s a need to devise cost-effective synthesis methods and strategies for recycling MOFs, ensuring the sustainability of the entire process.

In conclusion, while MOF composite membranes offer a promising avenue for wastewater treatment, continuous research and innovation are essential to fully harness their potential and address the multifaceted challenges of wastewater management.

## Figures and Tables

**Table 1:** Structure and adsorption properties of different MOFs.

Modification Strategy	Material Performance Improvement	MOFs	Adsorption Property	References
Mental doping	Specific Surface Area	UiO-67: 1653 m^2^/g ^a^	RhB: 41.3 mg/g ^b^	[72]
		Ce-UiO-67: 1795 m^2^/g ^a^	RhB: 754.4 mg/g ^b^	
	Specific Surface Area	ZIF-8: 246.37 m^2^/g ^a^	MG: 80% ^c^	[73]
		ZIF-8@Fe/Ni: 919.05 m^2^/g ^a^	MG: 99% ^c^	
Hierarchical MOFs	Specific Surface Area	3DPCNF: 24.954 m^2^/g ^a^	Cr(VI): 94% ^c^ Pb(II): 97% ^c^	[81]
		Co-Al-LDHFe_2_O_3_/3DPCNF: 49.66 m^2^/g ^a^	Cr(VI): About 40% ^c^ Pb(II): About 40% ^c^	
Etching	Specific Surface Area	UiO-66: 1353.1 m^2^/g ^a^	PA: 174 mg/g ^b^	[85]
		UiO-66-0.2TCA: 1419.3 m^2^/g ^a^	PA: 205.4 mg/g ^b^	
Functionalization	Active site	MIL-101	Pb (II): 15.7 mg/g ^b^	[96]
		ED-MIL-101	Pb (II): 87.64 mg/g ^b^	
Shape adjustment	Active site	ZIF-8	Phosphate: 54.82 mg/g ^b^	[101]
		2D ZIF-L	Phosphate: 75.18 mg/g ^b^	

Annotation: ^a^ is the specific surface area value; ^b^ is the adsorption capacity; ^c^ is the Removal rate.

**Table 2:** Applications of MOF compositions and MOF-membrane composite.

	Names of the Explored Composite	Material Performance Improvement	Applications	Performance	References
MOF-based composites	Fe_3_O_4_/ZIF-8	Specific Surface Area	Remove Pb^2+^	Adsorption capacity: 719.42 mg/g	[74]
	LDH@MOF-76	Active site	Remove U(VI)	Adsorption capacity: 433.9 mg/g	[86]
	HAP/ZIF-67	Active site	Remove U(VI)	Adsorption capacity: 453.1 mg/g	[87]
	Fe_3_O_4_@ZIF-8	Active site	Remove U(VI)	Adsorption capacity: 539 mg/g	[88]
	PTA@MIL-53(Fe)	Active site	Remove tetracycline hydrochloride	Adsorption capacity: 1250 mg/g	[89]
	Cu(tpa)/GO	Active site	Remove Mental ions (Cu^2+^, Mn^2+^, Cd^2+^, Zn^2+^, Fe^3+^ and Pb^2+^ )	Adsorption capacity: 235, 150, 53, 89, 78, 37 mg/g	[91]
	PEI@UiO-66-NH_2_	Active site	Remove Pb (II) and methyl orange (MO)	Adsorption capacity: 692.80, 497.51 mg/g	[92]
MOF composite membranes	MOF TFC FO membrane	Permeability	Desalination performance	Seawater fluxes: 34 L/m^2^h	[121]
	ZIF-8/PVDF TFC membrane	Permeability	Desalination performance	Seawater fluxes: 6 Kg/m^2^h	[122]
	HKUST-1@mPES MMM	Permeability	Remove bovine serum albumin	Pure water permeability: 490 L·m^−2^·h^−1^·bar^−1^; bovine serum albumin rejection rate: 96%	[123]
	MAF-9/PDMS membrane	Selectivity	Remove butanol	Butanol flux: 378 g·m^−2^·h^−1^; water flux: 471 g·m^−2^·h^−1^	[131]
	Cu-BTC/CA membrane	Selectivity	Remove pesticides	The adsorption capacity of dimethoate: 282.3–321.9 mg/g	[132]
	PDA/MOF-TFN membrane	Selectivity	Remove salt rejection and heavy metal ion removal	Salt reverse salt flux: about 3.5 gMH; removal rate of heavy metal ion (Cd^2+^, Ni^2+^ and Pb^2+^) > 94%	[134]
	UiO-66-NH_2_ membrane	Selectivity	Mg^2+^ and Li^+^ separation in brine	Cation separations (Na^+^/Mg^2+^ > 200 and Li^+^/Mg^2+^ > 60)	[139]
	PAN/PEI/MIL membrane	Selectivity	Remove insoluble emulsified oils	Removal rate of insoluble emulsified oils: 99%	[141]
	GO@Al-MOF membrane	Stability	Remove dye	Congo red rejection rate: 99%; water permeability 51.6 L·m^−2^·h^−1^·bar^−1^	[142]

## Data Availability

Data will be made available on request.
